# Development of Fluorescence Polarization Immunoassay for Imidacloprid in Environmental and Agricultural Samples

**DOI:** 10.3389/fchem.2020.615594

**Published:** 2020-12-02

**Authors:** Liangliang Zhou, Jiachuan Yang, Zhexuan Tao, Sergei A. Eremin, Xiude Hua, Minghua Wang

**Affiliations:** ^1^Department of Pesticide Science, State & Local Joint Engineering Research Center of Green Pesticide Invention and Application, Ministry of Education, College of Plant Protection, Nanjing Agricultural University, Nanjing, China; ^2^Chemical Faculty, M.V. Lomonosov Moscow State University, Moscow, Russia

**Keywords:** imidacloprid, fluorescence polarization immunoassay, fluorescent tracers, pesticide residue, high throughput detection

## Abstract

A fluorescence polarization immunoassay (FPIA) for the determination of imidacloprid (IMI) was developed with advantages of simple operation and short assay time. The haptens of IMI, acetamiprid (ACE), and thiamethoxam (THI) were conjugated with fluorescein isothiocyanate ethylenediamine (EDF) and 4′-Aminomethyl fluorescein (AMF), respectively, to prepare six fluorescence tracers. The conjugation of IMI hapten and EDF (IMI-EDF) was selected to develop the FPIA due to the largest fluorescent polarization value increase in the presence of anti-IMI monoclonal antibody. Under the optimum condition, the limit of detection, 50% inhibition concentration and detection range of the FPIA were 1.7, 4.8, and 1.7–16.3 μg/L, respectively. The cross-reactivities (CRs) with the analogs of IMI were negligible except for imidaclothiz with CR of 79.13%. The average recovery of spiked paddy water, corn and cucumber samples were 82.4–118.5% with the RSDs of 7.0–15.9%, which indicated the FPIA had good accuracy. Thus, the developed FPIA was a potential tool for the rapid and accurate determination of IMI in agricultural and environmental samples.

## Introduction

Imidacloprid (IMI) [1-6(chloro-3-pyridylmethyl)-N-nitroimidazo-lidin-2-ylideneamine] is one of the ultra-efficient neonicotinoid insecticides, which operates as a competitor to postsynaptic nicotinic receptors in a central nervous system of the insect. Currently, IMI has been extensively used in agricultural product in many countries because of its excellent insecticidal effectiveness (Lee et al., 2001). However, IMI shows high toxicity to honeybees (Rebecca et al., 2020; Wang et al., 2020) and its residues also have potentially hazardous for consumers and ecosystem (Ana et al., 2019; Zhang et al., 2020). Therefore, it is necessary to monitor the IMI residual in agricultural and environmental samples.

At present, the instrument-based methods, such as high-performance liquid chromatography (HPLC) (Carretero et al., 2003; Saeedeh et al., 2020) and gas chromatography-tandem mass spectrometry (Su et al., 2017; Massara et al., 2018), have been widely used for the determination of IMI. Compared with instrument, immunoassay, as a rapid detection technique, has been widely used for the detection of small molecules due to its advantages in simplicity, specificity, low consumption, and high sensitivity. There are also many immunoassays that have been established for the detection of IMI, such as enzyme-linked immunosorbent assay (ELISA) (Watanabe et al., 2004; Brian et al., 2009; Navarro et al., 2013), and immunochromatographic assay (ICA) (Xu and Xu, 2012; Fang et al., 2015; Yang et al., 2018). However, ELISAs require long incubation time and multi-step operation, and ICAs generally provide qualitative or semi-quantitative results. Fluorescence Polarization Immunoassay (FPIA), as a homogeneous immunoassay, has attracted more and more attention because of simple operation, short assay time and high throughput (Smith and Eremin, 2008; Yue et al., 2014), and has been used for determination of small molecular compounds (Nasir and Jolley, 2002; Shim et al., 2004; Chun et al., 2009; Mi et al., 2013). The general principle of FPIA for small molecule is that the reaction between fluorescent tracer (fluorescein labeled competing antigen) and antibody results in a change of fluorescence polarization (FP) value. With the increase of the concentration of analyte, the tracer bound to antibody would decrease, which leads to the decrease of the FP value. Compared with heterogeneous immunoassay, FPIA shows some valuable advantages, such as simple operation (one step), short assay time (10–20 min) and good reproducibility due to less interference from inner-filter effects (Anna et al., 2017; Elena et al., 2018; Zhang et al., 2018). Besides, the application and popularity of portable polarimeter makes FPIA show great potential in on-site detection.

In this paper, six fluorescent tracers were prepared by conjugation of haptens of IMI, acetamiprid (ACE), and thiamethoxam (THI) with fluorescein isothiocyanate ethylenediamine (EDF) and 4′-Aminomethyl fluorescein (AMF), respectively. A FPIA for the determination of IMI was developed by employing anti-IMI monoclonal antibody (mAb, 3D11B12E5) and IMI-EDF. The accuracy of the FPIA was evaluated by the detection of IMI in spiked and authentic samples, and validated by HPLC.

## Materials and Methods

### Reagents

Imidaclothiz (97.82%) was provided by Nantong Jiangshan Agrochemical and Chemicals Co., Ltd. (Jiangsu, China). IMI and its other structural analogs were purchased from Dr. Ehrenstorfer GmbH (Germany). The anti-IMI mAb (3D11B12E5) and IMI hapten were prepared as described previously (Yang et al., 2018). The haptens of ACE and THI were prepared as described previously (Wanatabe et al., 2001; Kim et al., 2003). *N,N*-Dimethylformamide (DMF), *N, N*′-Dimethylformamide (DCC) and 4′-Aminomethyl fluorescein (AMF) were purchased from Sigma-Aldrich Chemical Co., Ltd (St. Louis, USA). Ethylenediamine (EDF) was prepared was described previously (Ding et al., 2019).

### Instruments and Equipments

The fluorescence intensity and FP value were measured by Spectra Max M5 (Molecular Devices, Sunnyvale, CA, USA). An Agilent 1260 HPLC equipped with an ultraviolet detector (Agilent, Wilmington, DE, USA) was used to verify the accuracy of the FPIA. Milli-Q purified water was obtained from a Milli-Q purification system (Millipore, Bedford, MA, USA). Black microplates (96-well) (3650, Corning Costar Corporation, NY, USA) was used as a reaction vessel for the FPIA.

### Preparation of Fluorescent Tracers

The haptens of IMI, ACE and THI were conjugated with EDF and AMF to prepare the tracers for the development of the FPIA. The procedure was carried out according to the previous articles (Kolosova et al., 2003; Ma et al., 2016). Briefly, 40 μmol hapten was dissolved in 0.5 mL DMF containing 80 μmol DCC, the mixture was stirred overnight at room temperature. After centrifugation for 10 min at 10,000 rpm, the supernatant was collected. Then, 10 mmol fluorescein (AMF or EDF) and 10 μL of triethylamine were added to 150 μL aforementioned supernatant and the reaction was allowed to proceed for 4 h. The fluorescent tracers (IMI-EDF, IMI-AMF, ACE-EDF, ACE-AMF, THI-EDF, and THI-AMF) were purified by thin layer chromatography (TLC). TLC boards were deployed in a chromatography cylinder containing chloroform and methanol (4:1, v/v), until the liquid moves to the top of plate (Xu et al., 2011). The major yellow bands were collected and eluted with methanol. Meanwhile, the R_f_ values of the yellow bands were calculated.

### Procedure of FPIA

One hundred microliter IMI standard solutions (or matrix solutions) and 50 μL fluorescent tracer in borate saline buffer (BB) were added to non-binding black microplates. Then, 50 μL mAb 3D11B12E5 in BB was added to the microplates to measure the FP value by SpectraMax M5 with the excitation wavelength of 492 nm and the emission wavelength of 526 nm.

### Optimization of Assay Conditions

In this study, the experimental parameters (concentration of antibody, incubation time, organic solvent, ionic strength, and pH) were investigated to improve the sensitivities of the FPIA. Under 6,400-fold dilution of the tracer, the FP values of the FPIAs with serial concentrations of mAb 3D11B12E5 (from 0.07 to 2.14 mg/L) were detected in the absence of analyte. When the FP value reached 50–80% of the FP_max_, the mAb concentration was desirable. The FPIA was used to detect IMI under the varying incubation time (from 0 to 15 min) and the serial working buffer with difference methanol content (0, 5, 10, 20, 30, and 40%), ionic strength (0.1, 0.2, 0.3, 0.4, 0.5, and 0.6 mol/L) and pH (4.4, 5.4, 6.4, 7.4, 8.4, and 9.4). The parameters that made the FPIA showed the lower IC_50_ and higher FP_max_/IC_50_ values were desirable.

### Specificity

A series of IMI analogs standard solutions were prepared and analyzed by the FPIA. The FPIA standard curves for different analogs were established to obtain 50% inhibition concentration (IC_50_). The IC_50_ values produced by analogs were used to calculate the cross-reaction (CR) according to the following formula:

$$\begin{array}{l} \text{CR}\left(\text{\%}\right)=\left[\text{I}{\text{C}}_{50}\left(\text{IMI}\right)/\text{I}{\text{C}}_{50}\left(\text{analog}\right)\right]\times 100 \end{array}$$

### Analysis of Spiked Samples

The IMI-free samples (corn, cucumber and paddy water, confirmed by HPLC) were collected from a farm in Nanjing, China, which were spiked with IMI at the final concentrations of 100, 500, and 1,000 μg/kg for corn and cucumber, final concentrations of 10, 50, and 100 μg/L for paddy water. The paddy water samples were filtered and directly analyzed using FPIA after mixing with an equal volume of 2× BB buffer. For other spiked samples, 10 g homogenized samples were weighed and extracted with 20 mL 80% methanol-BB. After vortexing for 5 min and ultrasonic for 10 min, the supernatants were separated by centrifugation for 10 min at 4,000 rpm, and adjusted to 25 mL. The concentrations of IMI in the spiked samples were analyzed by FPIA after appropriate dilution.

### The Correlation of FPIA With HPLC

Seven samples containing incurred residues (two paddy waters, two corns and three cucumbers were collected from a local farm in Nanjing, China) were simultaneously analyzed by HPLC and FPIA. The pretreatments of the samples for the FPIA were the same as the spiked samples described above. For HPLC, 20 mL paddy water was extracted by 40 mL acetonitrile by vortexing for 10 min. Subsequently, 5 g sodium chloride was added to stratify acetonitrile and water. The half of acetonitrile (20 mL) was transferred and evaporated to dryness with a rotary evaporator at 40 °C. The other samples (20 g) were extracted by vigorously shaking for 1 h with 50 mL of 80% acetonitrile aqueous solution. After filtration, the solution was then stratified with 5 g sodium chloride. And then, 20 mL of acetonitrile were transferred and evaporated to dryness with a rotary evaporator at 40°C. The extracts were dissolved with 5 mL of acetonitrile:water (70:30, v/v) and the concentrations of IMI were detected by HPLC with a Eclipse plus C18 column (4.6 mm × 250 mm × 5 μm). A mixture of acetonitrile:water (70:30, v/v) was used as the mobile phase at a flow rate of 1.0 mL/min at 30°C. The detection wavelength was 270 nm and the injection volume was 20 μl (Yang et al., 2018).

## Results and Discussion

### Selection of Fluorescent Tracers

**Figure 1:**
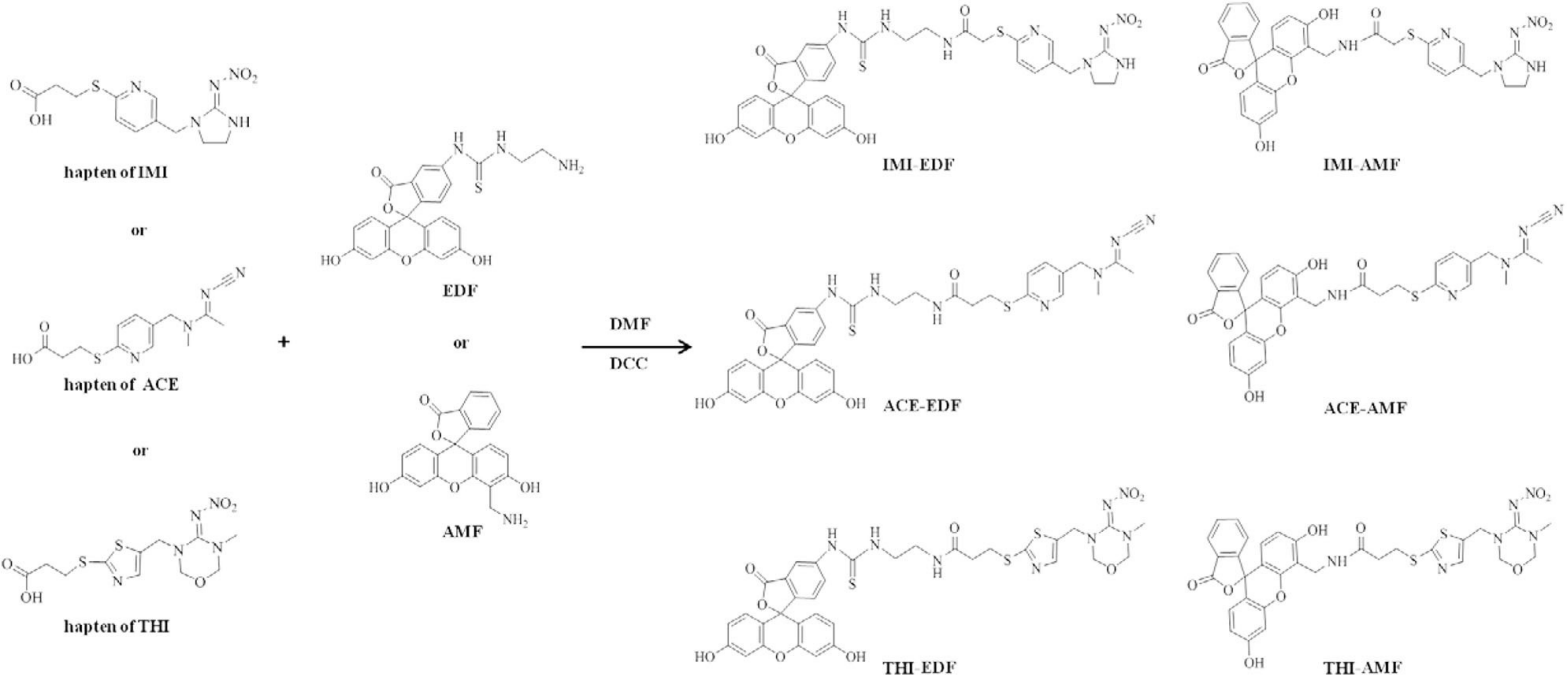
Synthesis of fluorescent tracers.

Three haptens of IMI, ACE and THI were conjugated with EDF and AMF, respectively (Figure 1), which yielded six fluorescent tracers (IMI-EDF, IMI-AMF, ACE-EDF, ACE-AMF, THI-EDF, and THI-AMF). The tracers were purified using TLC, the yellow bands with R_f_ = 0.6 for IMI-EDF, R_f_ = 0.7 for IMI-AMF, R_f_ = 0.6 for ACE-EDF, R_f_ = 0.7 for ACE-AMF, R_f_ = 0.5 for THI-EDF and R_f_ = 0.6 for THI-AMF were collected (Supplementary Figure 1). The tracers were diluted to fluorescence intensity near 100. The dilution times were 1,600, 6,400, 6,400, 6,400, 3,200, and 25,600 for IMI-AMF, IMI-EDF, THI-AMF, THI-EDF, ACE-AMF, and ACE-EDF, respectively (Supplementary Table 1). Under the dilutions, the tracers prepared by EDF (IMI-EDF, THI-EDF, and ACE-EDF) could bind with mAb 3D11B12E5 to increase the FP values, but the FP values of the tracers prepared by AMF (IMI-AMF, THI-AMF, and ACE-EDF) were not changed (Table 1). This result indicated that EDF was more suitable for the preparation of tracers, probably because EDF has a longer spacer. As expected, IMI-EDF showed the largest FP value increase, so it was chosen to develop the FPIA.

**Table 1:** The polarization of tracers.

**Solutions**		**Tracers**					
		**IMI-AMF R_**f**_ = 0.7,** **3,200**	**IMI-EDF R_**f**_ = 0.6,** **25,600**	**THI-AMF R_**f**_ = 0.6,** **6,400**	**THI-EDF R_**f**_ = 0.5,** **6,400**	**ACE-AMF R_**f**_ = 0.7,** **1,600**	**ACE-EDF R_**f**_ = 0.6,** **6,400**
BB	Fluorescence value	106.03	64.98	81.24	84.12	129.78	123.3
	Polarization value	44.52	64.92	46.51	51.38	39.29	43.90
BB with antibody	Fluorescence value	106.02	39.95	82.55	80.11	124.5	109.62
	Polarization value	44.83	149.78	47.33	63.45	39.32	60.09

### Optimization of the FPIA

As shown in Table 2, the FP value increased with the increasing concentration of mAb 3D11B12E5. When the concentration of mAb was 0.13 mg/L, the FP value was 134.75, which was in the range of the 50 to 80% of combination. The IC_50_ values of the FPIA with different incubation time were in the range of 5.75 and 5.89 ng/mL (Supplementary Figure 2A), which were no significant difference. Therefore, the FP values could be determined immediately after addition of mAb 3D11B12E5. The organic solvent is essential reagent in extraction and dissolution of pesticide, which usually shows great influence on immunoassays. Methanol is commonly used in immunoassays because of its relatively weak effect on immunoreactions between antibody and antigen. As shown in Supplementary Figure 2B, with the increase of methanol content, the IC_50_ values increased and the mPmax/IC_50_ values decreased. Finally, the maximum tolerance to methanol of the FPIA was 5%. The optimal concentration of Na^+^ and pH were 0.1 mol/L and 7.4, respectively, because the FPIA showed the highest FP_max_/IC_50_ (Supplementary Figures 2C,D).

**Table 2:** The polarization of antibody concentration.

**Antibody (mg/L)**	**2.14**	**1.07**	**0.54**	**0.27**	**0.13**	**0.07**	**Buffer**
Polarization value	235.50	228.69	222.95	163.87	134.75	115.28	61.79
Fluorescence value	41.00	39.92	39.17	49.01	51.21	54.24	74.73

### Sensitivity and Specificity

**Figure 2:**
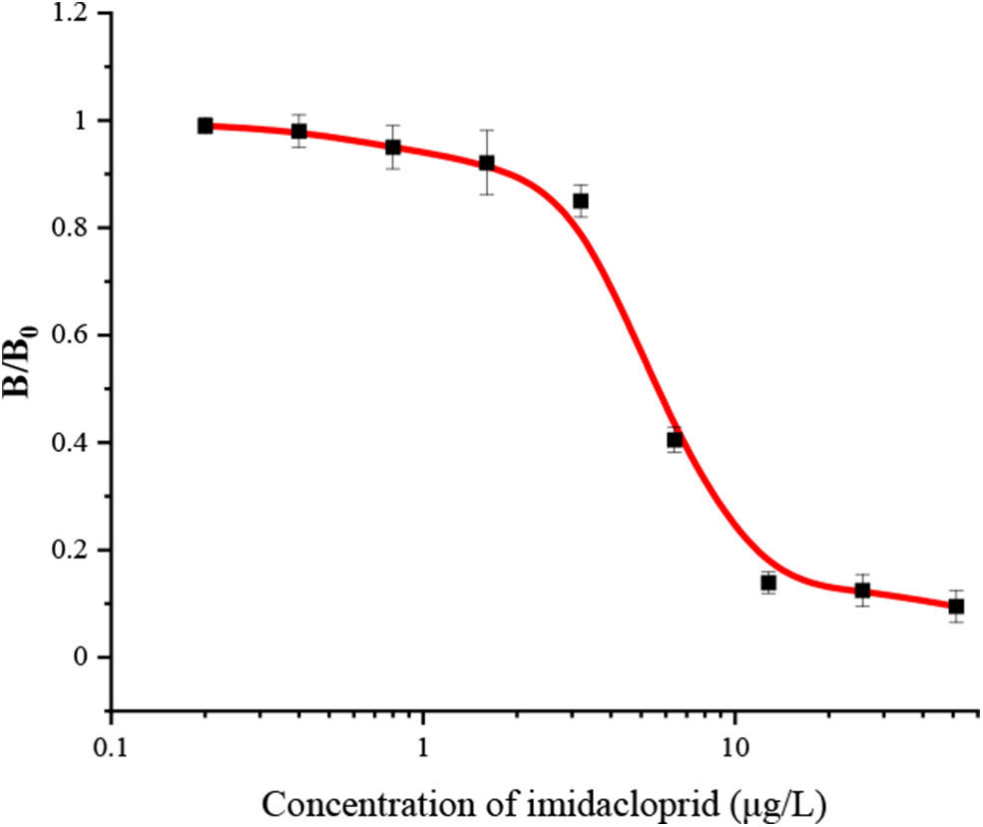
The standard curve of FPIA for IMI.

The FPIA for IMI was developed in a competitive format. With the increase of concentration of IMI, the IMI-EDF bound to mAb 3D11B12E5 would decrease, which resulted in the decrease of FP value. Under the optimal conditions, the standard curve of the FPIA for IMI was shown in Figure 2. The IC_50_, limit of detection (LOD, IC_10_) and linear range were calculated as 4.8, 1.7, and 1.7–16.3 μg/L, respectively. Compared with the reported articles, the FPIA showed higher sensitivity (IC_50_) than the enzyme-linked immunosorbent assay (ELISA) with IC_50_ of 17.3 ng/mL (Jae et al., 2001) and inner filter effect (IFE) immunoassay with SC_50_ (the concentration recovering 50% saturation of the signal) of 18.7 μg/L (Si et al., 2018). Although the FPIA had lower sensitivity than the fluorescence-based immunoassay (FIA) with IC_50_ of 1.3 ng/mL (Li et al., 2019) and the lateral flow immunoassays (LFIAs) with IC_50_ values of 0.13 and 0.14 ng/mL (Tan et al., 2020), it had advantages of simpler operation and high-throughput test. Importantly, the maximum residue limits (MRL) of IMI on agricultural products are in the range of 0.05 to 10 mg/kg in China, for example, the MRLs of IMI are 0.05 mg/kg for corn and 1 mg/kg for cucumber. Besides, and the range of MRLs are 0.01 to 50 mg/kg in Food and Agriculture Organization of the United Nations (FAO), and the MRLs for cereal grains and cucumber are 0.05 and 1 mg/kg, respectively. The sensitivity of the FPIA could meet the requirements for the detection of IMI under an appropriate pre-treatment.

The CRs of the FPIA for the analogs were negligible (≤7.6%) except for imidaclothiz with CR of 79.1% (Table 3), because they both have nitro-dihydroimidazol-amine group, which was an important part for antibody recognition. According to the reported articles, most immunoassays for IMI showed CR with imidaclothiz. Si et al. (2018) reported an IFE immunoassay showed 90.3, 32.7, and 32.8% CRs for imidaclothiz, thiacloprid, and chothianidin, respectively. Guo et al. (2021) developed a fluorescence resonance energy transfer (FRET) immunoassay for IMI, which had 74.4% CR with imidaclothiz.

**Table 3:** Cross-reactivity of IMI toward some of its analogs by FPIA.

**Compound**	**Structure**	**IC_**50**_ (μg/L)**	**CR (%)**
Imidacloprid	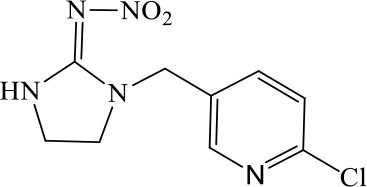	4.8	100.0
Imidaclothiz	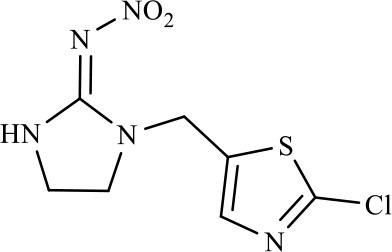	6.1	79.1
Clothianidin	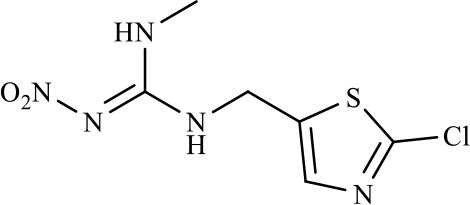	64.4	7.6
Thiacloprid	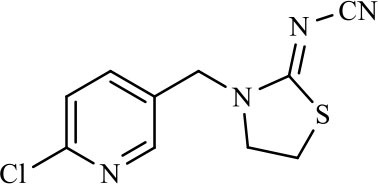	125.8	3.9
Acetamiprid	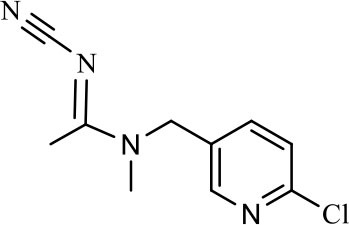	166.3	2.9
Nitenpyram	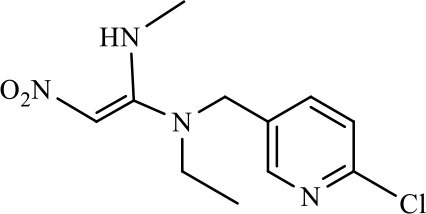	250.1	1.9
Dinotefuran	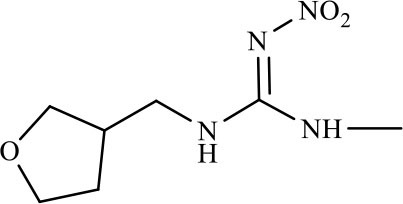	458.9	1.1
Thiamethoxam	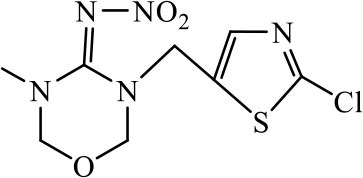	>10,000	<0.05

### Matrix Effect and Recovery of Spiked Samples

The sample matrix could affect the accuracy of the immunoassay and is usually removed by dilution with buffer. As shown in Supplementary Figure 3, the matrix interference of corn and cucumber could be eliminated after 16-fold dilution, and paddy water was 2-fold dilution at least, because the standard curve prepared by the diluted matrix were closest to the standard curve prepared by buffer. Under the dilutions, the average recoveries of FPIA for spiked samples were range in 82.4–118.5% with relative standard deviation (RSD) of 7.0–15.9% (Table 4).

**Table 4:** Recovery of IMI in spiked samples.

**Samples**	**Spiked concentration** **(μg/L or μg/kg)**	**Recovery (%)**	**RSD (%)**
Paddy water	10	89.1	11.2
	50	103.0	7.0
	100	118.5	12.5
Corn	100	86.0	15.9
	500	84.1	10.9
	1,000	108.5	14.1
Cucumber	100	82.4	9.0
	500	83.0	8.3
	1,000	108.5	13.2

### The Validation of FPIA With HPLC

**Figure 3:**
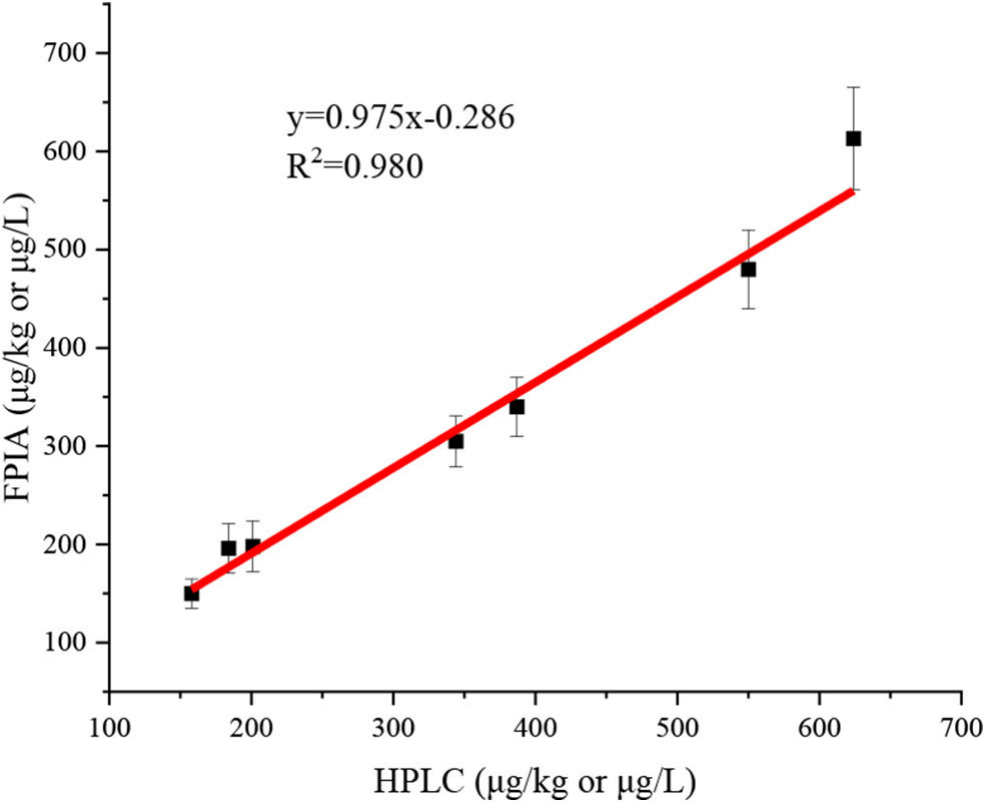
Correlation between FPIA and HPLC for the concentrations of IMI in authentic samples.

Seven authentic samples were tested by HPLC and FPIA simultaneously. There were good correlations between FPIA and HPLC, because the slope value of correlation curve was very close to 1 (y = 0.975x - 0.286, *R*^2^ = 0.980) (Figure 3). These results indicated that the FPIA were reliable for quantitative detection of IMI in authentic samples.

## Conclusions

In this study, serial tracers were prepared by conjugation of IMI, ACE, and THI haptens with EDF and AMF. The tracer of IMI-EDF was employed to develop a FPIA for IMI because of the largest FP value increase in the presence of mAb. The FPIA was a homogeneous one-step assay that does not require incubation and washing. The samples can be tested directly after simple processing. The LOD, IC_50_ value and the linear range of the FPIA were 1.7, 4.8, and 1.7–16.3 μg/L, respectively. The FPIA showed the CR of 79.1% for imidaclothiz. In addition, the results of the FPIA for the authentic samples were in good agreement with those of HPLC. Therefore, the FPIA can be used to detect IMI in agricultural and environmental samples. Besides, the FPIA also can combine with the portable polarimeter to realize quickly and on-site detection.

## Data Availability Statement

The original contributions presented in the study are included in the article/Supplementary Material, further inquiries can be directed to the corresponding author/s.

## Author Contributions

LZ: conceptualization, methodology, software, investigation, formal analysis, and writing—original draft. JY: preparation of fluorescent tracers, formal analysis, and supervision. ZT: investigation, software, and original draft. SE: review, editing, visualization, and supervision. XH: validation, formal analysis, visualization, and supervision. MW: resources, writing—review and editing, supervision, data curation, and project administration. All authors contributed to the article and approved the submitted version.

## Conflict of Interest

The authors declare that the research was conducted in the absence of any commercial or financial relationships that could be construed as a potential conflict of interest.
