# Supplementary material for: Development of Fluorescence Polarization Immunoassay for Imidacloprid in Environmental and Agricultural Samples
Source: Front Chem. 2020 Dec 2;8:615594. doi: 10.3389/fchem.2020.615594 (PMC7738439; doi:10.3389/fchem.2020.615594)
Supplement: Supplementary file 1 [file Data_Sheet_1.docx]

**Development of fluorescence polarization immunoassay for imidacloprid in environmental and agricultural samples**

Liangliang Zhou^1^, Jiachuan Yang^1^, Zhexuan Tao^1^, Sergei A. Eremin^2^, Xiude Hua^1^ and Minghua Wang ^1*^

^1^ *Department of Pesticide Science, College of Plant Protection, Nanjing Agricultural University, State & Local Joint Engineering Research Center of Green Pesticide Invention and Application, Ministry of Education, Nanjing 20095, P. R. China*

^2^*Chemical Faculty, M.V. Lomonosov Moscow State University, Moscow 119991, Russia*

^*^*Corresponding author. Tel.: +86 25 84395479. E-mail address:* [*wangmha@njau.edu.cn*](mailto:wangmha@njau.edu.cn).

**Supplementary data**

**Table S1** The fluorescence value of tracers

**Figure S1** The purification results by TLC

**Figure S2** Effects of reaction time, methanol content, ionic strength, pH values on the FPIA.

**Figure S3** The evaluation of matrix effects for various samples on the FPIA.

Table S1. The fluorescence value of tracers

| Dilution time | IMI-AMF R_f_=0.7 | IMI-EDF R_f_=0.6 | THI-AMF R_f_=0.6 | THI-EDF R_f_=0.5 | ACE-AMF R_f_=0.7 | ACE-EDF R_f_=0.6 |
| --- | --- | --- | --- | --- | --- | --- |
| 400 | 452.67 | 1516.55 | 1639.67 | 1789.97 | 1045.62 | 5825.73 |
| 800 | 231.66 | 610.97 | 827.14 | 941.88 | 554.78 | 2786.53 |
| 1600 | 106.09 | 399.95 | 423.03 | 463.99 | 278.05 | 1338.8 |
| 3200 | 52.32 | 168.05 | 217.91 | 236.67 | 138.4 | 721.27 |
| 6400 | 28.48 | 97.78 | 106.83 | 109.47 | 69.97 | 343.41 |
| 12800 | 15.48 | 47.82 | 54.79 | 57.67 | 34.41 | 178.5 |
| 25600 | 8.73 | 27.06 | 27.98 | 28.86 | 18.97 | 88.03 |
| BB | 1.87 | 2.52 | 1.51 | 2.28 | 1.35 | 1.51 |


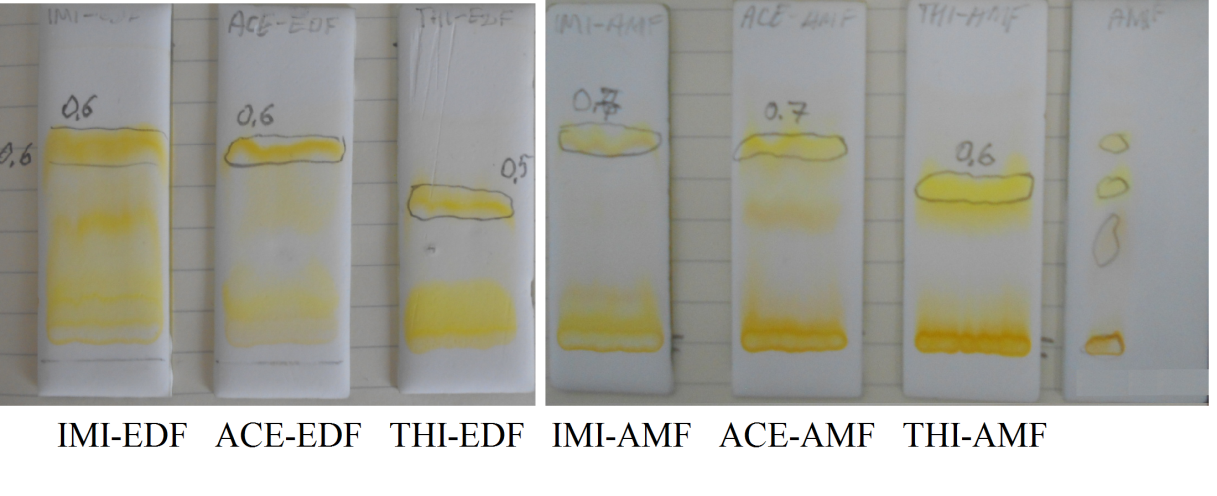


THI-AMF


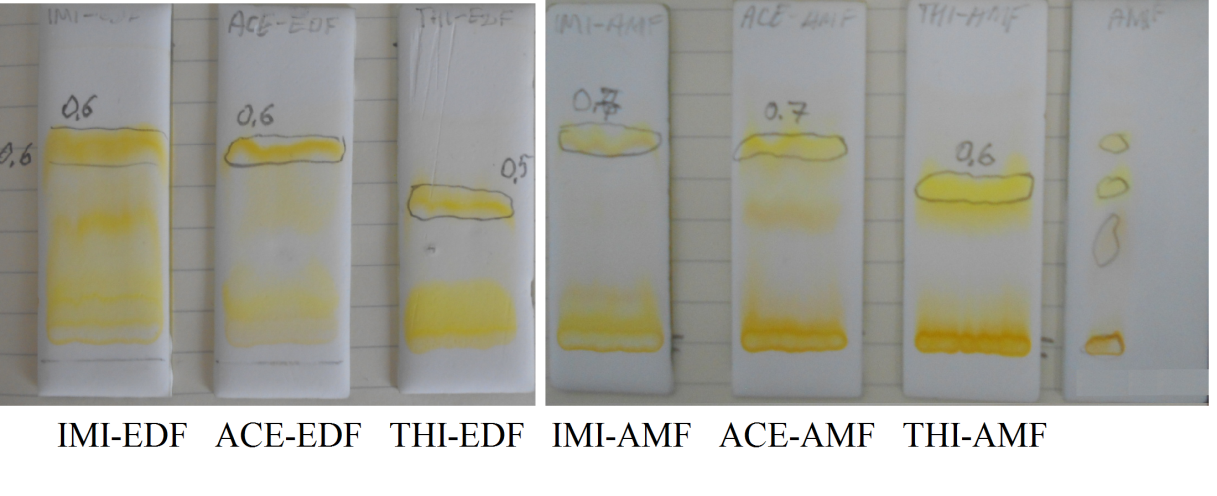


THI-EDF


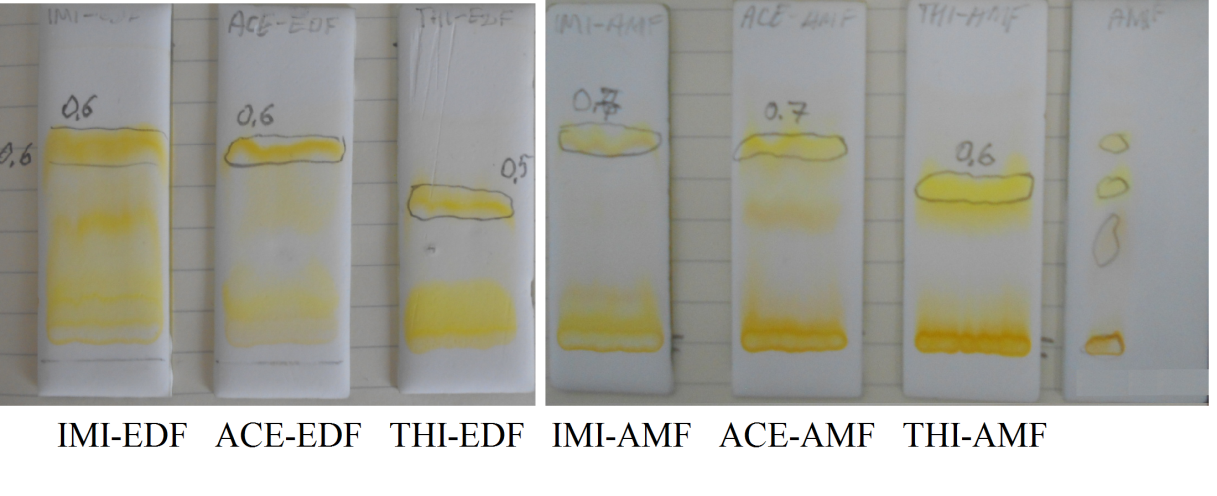


ACE-AMF


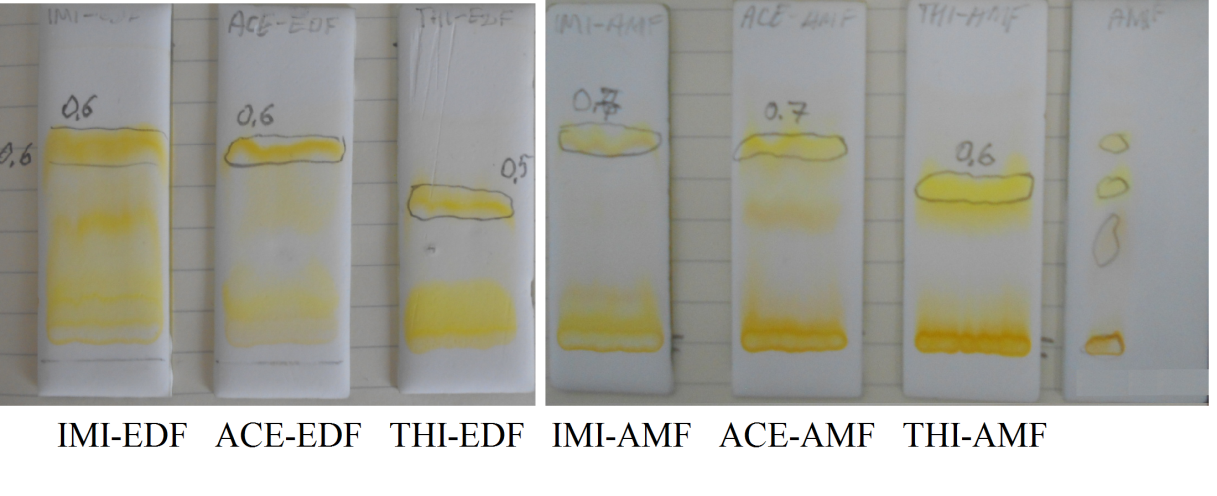


IMI-AMF


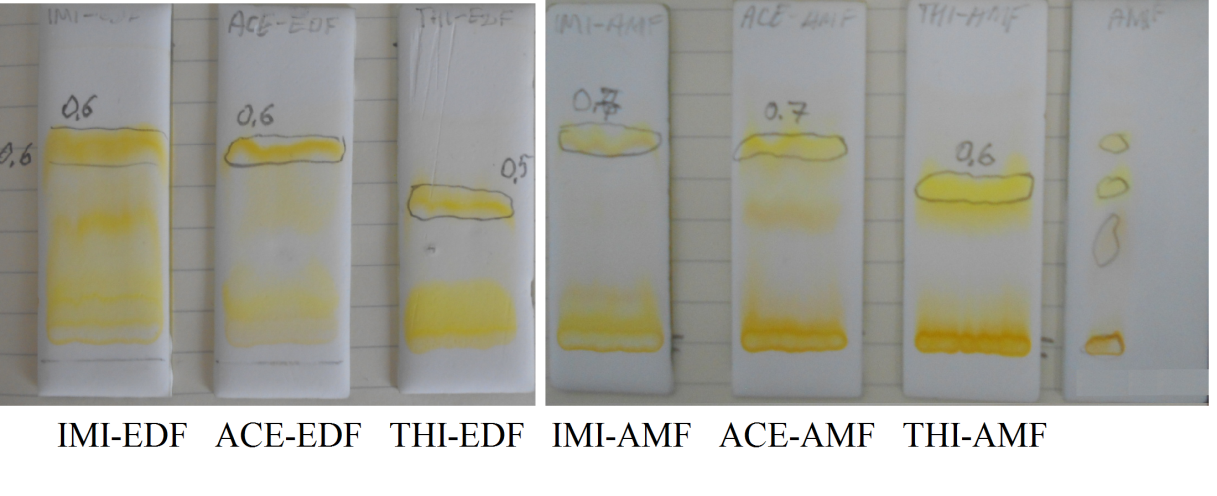


IMI-EDF


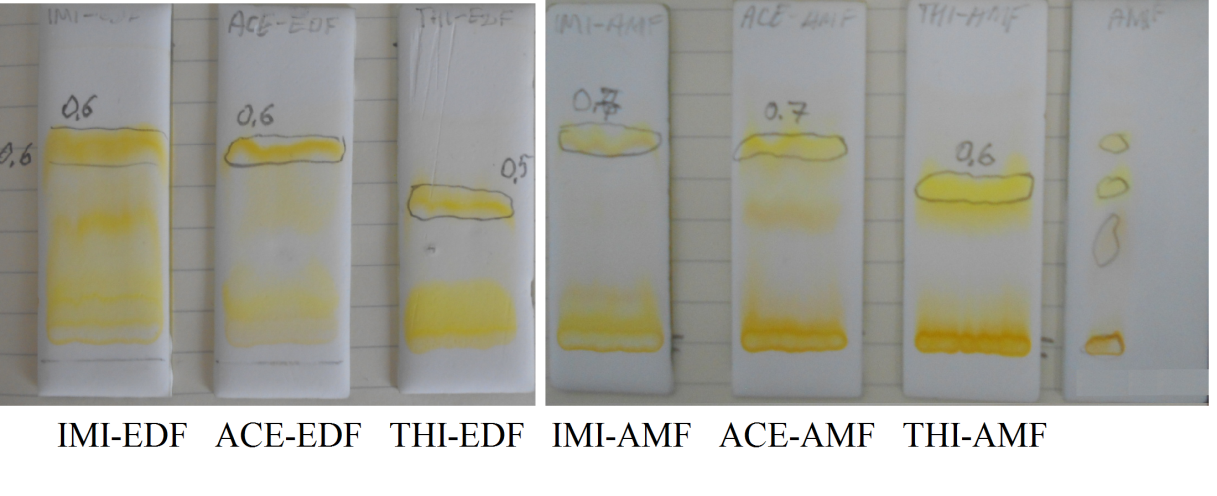


ACE-EDF

Figure S1. The purification results by TLC.

**
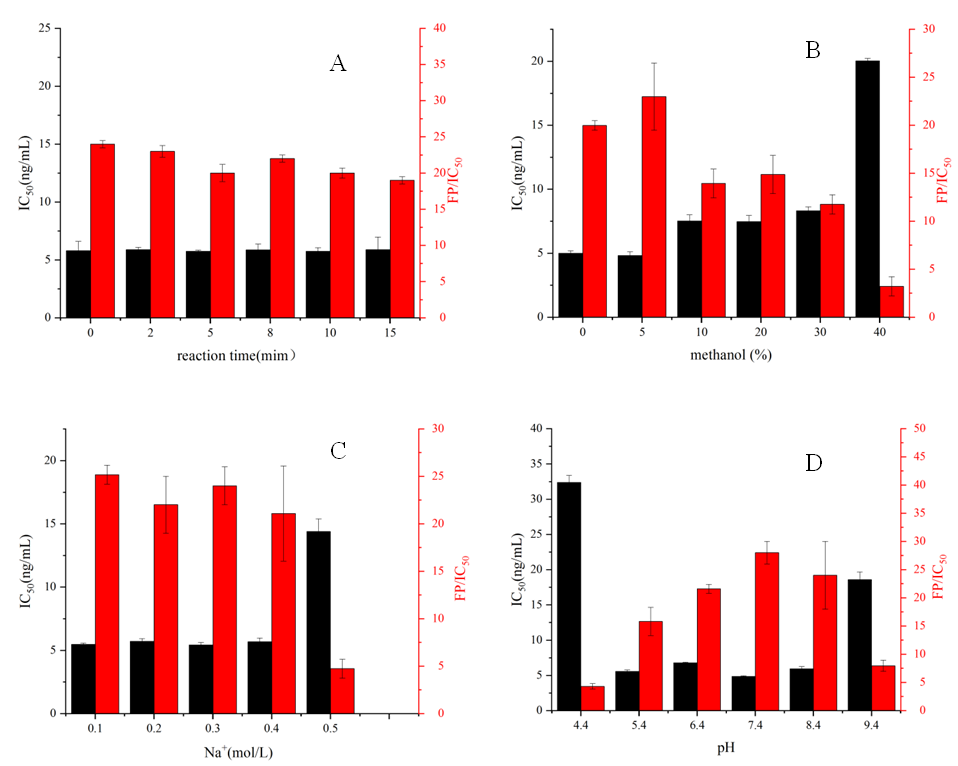
**

**Figure S2** Effects of reaction time, methanol content, ionic strength, pH values on the FPIA.

**
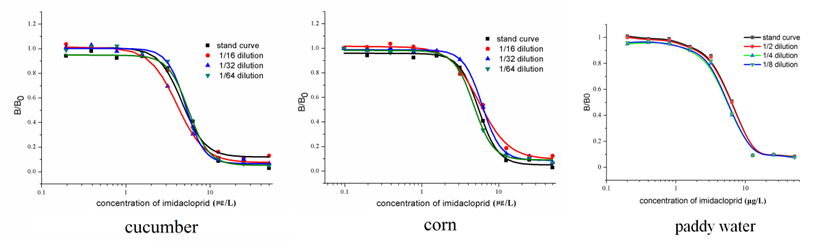
**

**Figure S3** The evaluation of matrix effects for various samples on the FPIA.
